# Health Risk Assessment of Dermal Exposure to Heavy Metals Content of Chemical Hair Dyes

**Published:** 2019-05

**Authors:** Fariba KHALILI, Amir Hossein MAHVI, Simin NASSERI, Masood YUNESIAN, Mehdi YASERI, Babak DJAHED

**Affiliations:** 1. Department of Environmental Health Engineering, School of Public Health, Tehran University of Medical Sciences, Tehran, Iran; 2. Center for Solid Waste Research, Institute for Environmental Research, Tehran University of Medical Sciences, Tehran, Iran; 3. Center for Water Quality Research, Institute for Environmental Research, Tehran University of Medical Sciences, Tehran, Iran; 4. Department of Environmental Health Engineering, Iranshahr University of Medical Sciences, Iranshahr, Iran

**Keywords:** Chemical hair dyes, Heavy metals, Risk assessment, Iran

## Abstract

**Background:**

Contamination of hair dyes to heavy metals can threaten consumer’s health. We investigated the concentrations of some important heavy metals in hair dyes and evaluates their non-carcinogenic effects.

**Methods:**

The most commonly used hair dyes were determined through questioners and 32 samples were collected from the market of Tehran in 2014. The concentration of 10 heavy metals (Fe, Ag, Co, Cr, Mn, Ba, Cd, Cu, Pb, and Al) was determined using ICP-MS. Based on the obtained data from distributed questionnaires and Monte Carlo simulation, the exposure to the evaluated heavy metals was estimated. Besides, using hazard quotient (HQ) and chronic hazard (HI), the risk of non-carcinogenic effects of investigated hair dyes consumption was specified.

**Results:**

Results indicated the average concentrations of Al, Ba, and Fe as 0.54, 0.86, and 1.19 mg kg^−1^ and those of Cd, Cu, and Pb as 0.45, 61.32, and 185.34 μg kg^−1^, respectively. Pb with HQ of 7.46e-4 had the highest risk and Fe with HQ of 3.4e–6 had the lowest level of risk. Among the investigated dyes, the ones made by Iran (HI=2.8e–4) and the dark brown color (HI=1.93e–4) had the highest level of risk among all the studied samples.

**Conclusion:**

Two indices of HI and HQ showed that heavy metal contents in the investigated samples had not probable non-carcinogenic risks for the consumers of these products.

## Introduction

Heavy metals are one of the most important environmental pollutants, some of which even at very low values could be dangerous for human health because they have long biological half-lives, are non-biodegradable, and some are toxic even at very low concentrations (1, 2). People are exposed to heavy metals daily through different pathways. Therefore, it could be one of the concerns for human health. These elements are released into the environment from both natural and anthropogenic activities (3), and the entrance of them to the human body can lead to different problems such as neurological and developmental disorders, reproductive disorders, cardiovascular diseases, skeletal, blood, immune, gastrointestinal disorders, renal problems, cancer, birth defects, allergic reactions, contact dermatitis, brittle hair and hair loss (4–6).

On the other hand, cosmetics products since the dawn of civilization are considered a part of routine body care (7). Direct contact with these materials and human’s skin causes the absorption of these materials into the human body (8). There are concerns regarding the presence of harmful chemicals in these products (9), hence, the assessment of different chemicals in personal care products is a public health issue since the use of these products could represent a possible source of human exposure to a variety of chemicals. Usually, during product processing, the cosmetic products are contaminated to heavy metals (10), and they are one of the sources of releasing of these elements (9). “The deliberate use of metals as active ingredients in cosmetic products is prohibited in most countries, but in the past, metals were utilized as ingredients of cosmetics, for example, the addition of the mercury, lead acetate in progressive hair dye and mercuric sulfide in a number of tattoo pigments” (4). Nowadays contamination to the heavy metals usually does exist in cosmetic products due to their ubiquitous natures of these elements.

Among different cosmetic products, hair dye is one of the most common products in the market. Skin is the direct way for entering the components of hair dyes (11). Hence, this kind of cosmetic and its ingredients have to be safe under the conditions of normal use and must be thoroughly evaluated prior to marketing. Hence, some studies, in the market of different countries, surveyed the concentration of heavy metals in both natural and chemical hair. For example in Iraq, the concentration of Lead, Copper, Iron, and cadmium were investigated in the range of 0.41–0.91, 0.26–0.31, 0.64–1.36 and 0.11–0.16 ppm respectively (12). Furthermore, in another study on natural hair dye in Nigeria, the levels of Cu, Pb, Cr, Ni and Zn were reported in the range of <0.03–20.5, <0.03–3.5, <0.1–9, 1.33–8.4 and <0.03–298 ppm, respectively, besides they found the concentration of Al, Cd, and Co under detection limit (13). Moreover, the levels of Pb, Cd, and Ni were investigated in both natural and synthetic hair dyes sold in the market of Turkey; the concentrations of Pb, Cd, and Ni found in synthetic hair dyes in the ranges of LOD-0.56 mg g–1, LOD-0.011 ng g–1 and 0.030–0.37 mg g–1, respectively, whereas those in the two natural hair dye were 0.60–0.93 mg g–1, 0.033–0.065 ng g–1 and 0.49–1.06 mg g–1, respectively (5). Unfortunately, there has been no study on the concentration levels of these harmful elements in available chemical hair dyes in the market of Iran.

Hence, in the current study, we determined the concentration of some heavy metals in the most widely used brands of chemical hair dyes in the market of Tehran, and then, we estimated the exposure levels of female residents of Tehran to these elements; and subsequently, the related non-carcinogenic health risk to the consumption of these products was evaluated.

## Materials and Methods

### Sampling

To determine the concentration of some important heavy metals in chemical hair dyes, in 2014, 8 widely used brands were selected from market of Tehran and, in each brand, 4 types of colors (black, blonde, light brown, and dark brown) were selected. The sampled brands were made by Iran, Spain, Italy, and Germany, although some of these brands probably were fake brands. The brands were gathered from different stores in the market of Tehran City randomly.

### Sample pretreatment and analysis

About 5 ml of HNO_3_ (nitric acid) was added to 1 gr of each sample. After heating for 4 h at 85 °C the sample was cooled down at room temperature and 1 ml of peroxide hydrogen 30% was added and it was injected to couple plasma-mass inductively spectrometry (ICP-MS) in order to determine the concentration of Fe, Ag, Co, Cr, Mn, Ba, Cd, Cu, Pb, and Al (14). The concentration of Ag, Co, Cr, and Mn was less than the detection of limit (LOD) in twenty percent of the samples; hence we neglected these elements for analysis. Detection limit for the metals such as Ba, Cd, Ag, and Cr was 0.05 ppb and this amount for Cu and Co was 0.1 ppb, for Fe and Mn was 0.1 ppm, and for Al and Pb was 1 ppb and 0.01 ppm.

### Exposure assessment and risk characterization

In order to evaluate exposure levels to heavy metals due to hair dyes consumption, 370 questionnaires were distributed in female citizens of Tehran and some key parameters such as weight, age of starting the use of chemical hair dyes, and frequency of hair dye consumption was questioned. Besides, Eq.1 was used to determine the heavy metal intake through skin exposure to hair dyes (15).

[1] $$D_{\mathrm{dermal}}=\frac{C\times \text{SA}\times \text{SL}\times \text{ABS}\times \text{EF}\times \text{ED}}{\mathrm{BW}\times \mathrm{AT}}\times 10^{-6}$$

Where D_dermal_ (mg kg^−1^day^−1^) is average daily intake of heavy metals through skin exposure to hair dyes, C (mgkg^−1^) is metal concentration in hair dye, SA (cm^2^) is the skin area of scalp, SL (mg cm^−2^) is skin absorption factor, ABC (unitless) is the depth absorption skin factor, EF (day year^−1^) is exposure frequency, ED (year) is exposure duration, BW (kg) is body weight, and AT (day) is average time. AT parameter is obtained using Eq.2 (16).

[2] AT=ED×365

Where ED (year) is the number of years that hair dyes are consumed. Besides, by considering the available information non-carcinogenic health risk effects were evaluated through Eq.3 (17–19).

[3] $$\mathrm{HQ}=\frac{D_{\mathrm{dermal}}}{\text{RfD}}$$

Where RfD (mg kg^−1^day^−1^) is the reference dose and HQ (unitless) is hazard quotient. Moreover, HI (chronic hazard index) is obtained by Eq.4, used to sum more than one HQ resulted from different elements or from different pathways.

[4] $$\mathrm{Chronic}\;\mathrm{Hazard}\;\mathrm{Index}=\sum_{k=1}^{n}\frac{D_{{\mathrm{dermal}}_{k}}}{{\mathrm{RfD}}_{k}}$$

### Uncertainty analysis

Risk assessment process is related to uncertainty that might arise from uncertainty in the measurement or estimation of parameters (20). Hence, for achieving a more accurate result, uncertainty analysis is necessary. In the current study, Monte Carlo uncertainty analysis was used to analyze uncertainty in exposure assessment. In this analysis, the stochastic behavior of the risk model is investigated using the probability distribution of inputs, random numbers, and statistical sampling methods (21). For this purpose, the distribution parameters of exposure model were evaluated using Easy Fit Professional Version 5.5 and, subsequently, uncertainty analysis was carried out by ver. 5.0.2.1 of the ModelRisk software (Vose Software, Belgium) and using 2000 iterations.

## Results

Findings from the distributed questionnaire showed that 92.19% of the participants used hair dye. In addition, about 19.5% of female citizens in Tehran started using this product from the age of 12 yr old, while most of the participants (34.7%) started using this product from the age of 19 yr old. Moreover, about half of the participants (49.5%) declared that they use chemical hair dyes. Furthermore, 4 colors (blonde, light brown, dark brown and black) had the highest consumption rate as 33.6%, 27.4%, 19.6%, and 14%, respectively. Moreover, based on the questionnaire, 8 brands were determined as the most widely used hair dyes; the countries which made those brands were as follows: Italy (19.7%), Iran (13.6%), Spain (8.3%), Iran (6.8%), Spain (6.8%), Iran (5.3%), Italy (5.3%), and Germany (5.3%). Four colors of each most widely used hair dye brands were purchased and were transferred to the laboratory for heavy metal measurement.

According to Table 1, the mean concentration of Al, Ba, Cd, Cu, Fe and Pb were determined 0.54±0.64, 0.86±1.43, 0.00045±0.0003, 0.061±0.1, 1.19±2.46 and 0.185±0.09 ppm, respectively. As Table 2 shows, the maximum concentration of Al was determined in second brand (1.59±1.43 ppm) made by Iran. Besides, the highest value of Ba, Cd, Cu, Fe and Pb was observed in brands of 7, 3, 1, 2 and 1, respectively. Furthermore, average concentrations of measured elements in terms of different colors are presented in Table 3. The maximum concentrations of Al, Ba, Cd, Cu, Fe and Pb were observed in light brown, dark brown, blond, black, black and blond, respectively.

**Table 1:** The average concentration, distribution type, distribution parameters and RFD for measured element

***Element***	***Mean±S.D***	***Median***	***Range***	***90% Cumulative Frequency***	***Distribution Mode***	***RfD_ing_ (mg/kg/d)***	***RfD_derm_ (mg/kg/d)***
Al (ppm) (n=32)	0.54±0.64	0.38	0.1–3.7	0.91	Lognormal	1	0.1
Ba (ppm) (n=32)	0.86±1.43	0.2	0.07–4.51	3.97	LogGamma	0.7	0.0049
Cd (ppb) (n=32)	0.45±0.3	0.4	0.1–1.2	0.95	Weibull	0.001	0.000025
Cu (ppb) (n=32)	61.32±100.59	18.7	2.6–334.7	285.4	Lognormal	0.04	0.012
Fe (ppm) (n=32)	1.19±2.46	0.39	0.17–11.58	2.28	Pareto	0.3	0.3
Pb (ppb) (n=32)	185.34±90.49	155.7	3.74–385.5	361.21	LogLaplace	0.0035	0.00052

**Table 2:** Concentration of measured metals in each brand of hair dyes

***Brands***	***Made by***	***Al (ppm) Mean±S.D***	***Ba (ppm) Mean±S.D***	***Cd (ppb) Mean±S.D***	***Cu (ppb) Mean±S.D***	***Fe (ppm) Mean±S.D***	***Pb (ppb) Mean±S.D***
Brand 1 (n=4)	Italy	0.42 ±0.43	-*	0.45±0.39	168.77±174.14	0.41±0.165	240.3±106.4
Brand 2 (n=4)	Iran	1.59 ±1.43	1.07±1.34	0.33±0.152	21.4±24.47	5.2±5.71	225.12±106.16
Brand 3 (n=4)	Spain	0.41 ±0.26	0.495±0.417	0.85±0.49	41.65±27.53	0.53±0.61	195.17±126.9
Brand 4 (n=4)	Iran	0.3 ±0.15	0.26±0.197	0.43±0.23	15.86±10.15	0.5±0.35	155.38±25.42
Brand 5 (n=4)	Spain	0.46 ±0.19	0.09±0.028	0.17±0.11	86.2±124.59	0.53±0.35	155.03±53.17
Brand 6 (n=4)	Iran	0.21 ±0.12	0.125±0.077	0.75±0.212	151.75±208.8	0.49±0.36	189.95±135.57
Brand 7 (n=4)	Italy	0.28 ±0.23	1.69±2.44	0.5±0.14	21.03±10.98	1.52±1.82	118.56±77.79
Brand 8 (n=4)	Germany	0.68 ±0.14	0.376±0.261	-*	13.9±13.71	0.32±0.1	203.23±54.19

* The number of samples below LOD were over 20%

**Table 3:** Concentration of measured metals in each color of hair dye

***Code color***	***Al (ppm) Mean±S.D***	***Ba (ppm) Mean±S.D***	***Cd (ppb) Mean±S.D***	***Cu(ppb) Mean±S.D***	***Fe(ppm) Mean±S.D***	***Pb(ppb) Mean±S.D***
Black (n=8)	0.51±0.32	1.08±1.67	0.483±0.285	82.8±114.8	2.29±3.98	169.9±106.59
Blond (n=8)	0.41±0.31	-*	0.5±0.37	69.28±117.61	0.44±0.42	228.7±100.36
Light Brown (n=8)	0.81±1.19	0.3±0.09	0.45±0.4	70.04±117.72	1.56±2.81	167.8±69.74
Dark Brown (n=8)	0.44±0.31	1.14±1.9	0.32±0.17	14.51±11.93	0.46±0.26	174.93±84.09

* The number of samples below LOD were over 20%

### Exposure assessment and risk characterization

**Fig. 1:**
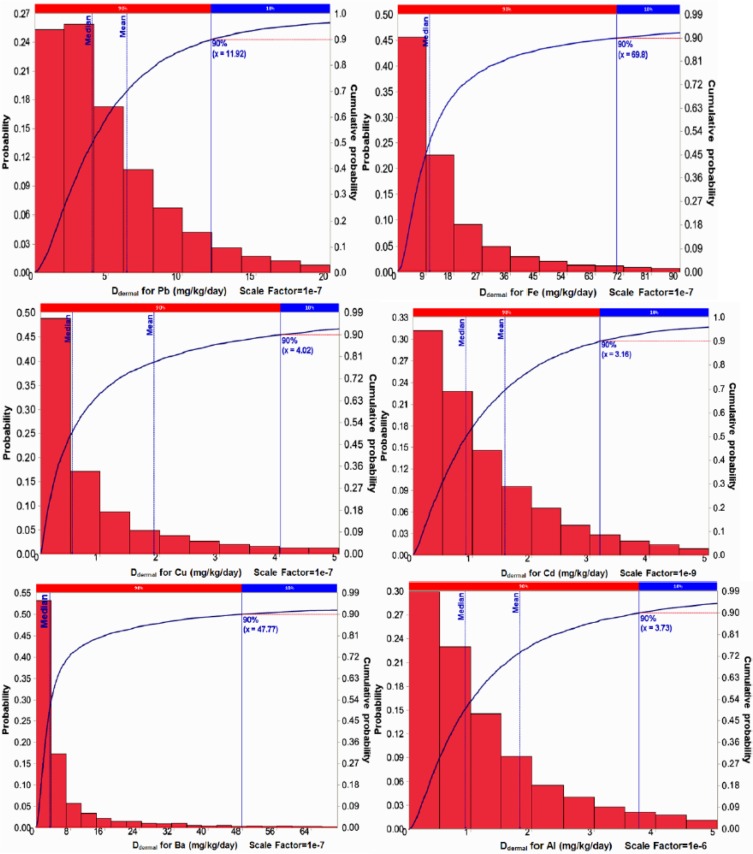
Probability distribution diagram D_dermal_ for Al, Ba, Cd, Cu, Fe, Pb

The average daily intake of heavy metals through skin exposure to investigated chemical hair dyes was evaluated using Monte Carlo simulation, for this propose, 20000 iterations were utilized to calculate Eq.1. The results are illustrated in Fig. 1.

**Fig. 2:**
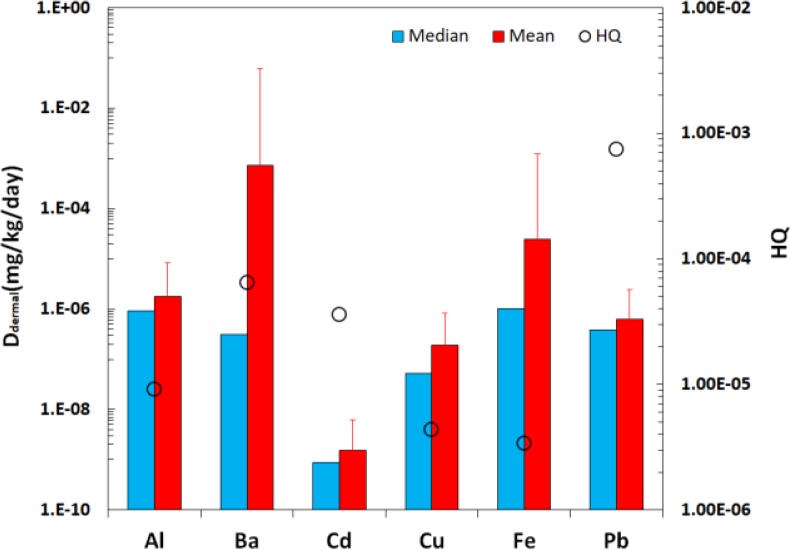
HQ values and mean and median of D_dermal_

**Fig. 3:**
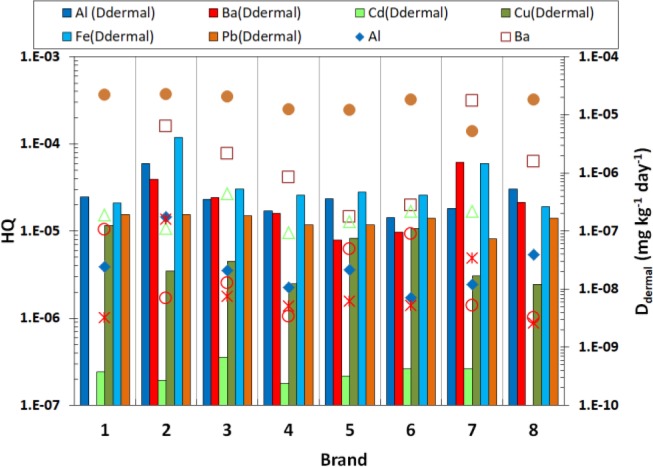
Levels of HQ and D_dermal_ in different brands

**Fig. 4:**
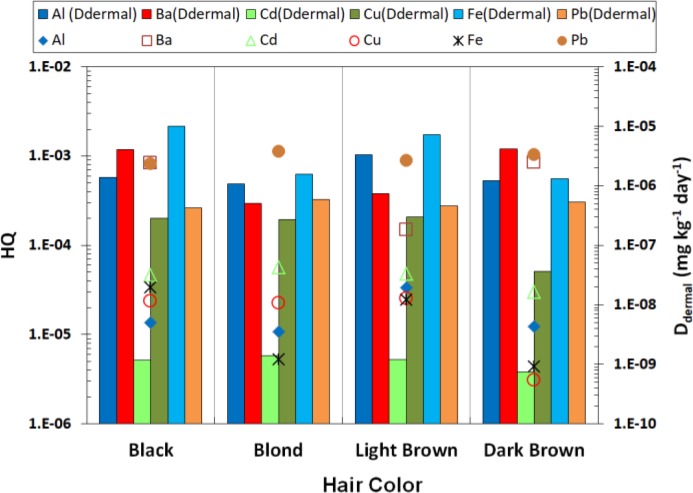
Levels of HQ and D_dermal_ in different colors

Central index of median in all the simulations had the highest probability than other central indices, hence, we used median of the D_dermal_ in order to determined risk characterization (Fig. 1). Besides, the comparison between the levels of daily intakes of evaluated elements (D_dermal_) is shown in Fig. 2. According to Fig. 2, maximum (7.29e–4 mg/kg/d) and minimum (1.55e–9 mg/kg/d) average of exposure to investigated elements were calculated for Ba and Cd, respectively. Moreover, D_dermal_ values of evaluated elements among different brands and different colors were presented in Figs. 3 and 4, respectively.

Besides, non-carcinogenic health risk of each element was investigated based on the calculated D_dermal_ (Fig. 2). Pb (7.46e–4) had the highest health risk among evaluated elements. Moreover, HQ was calculated for different brands and colors and the results are depicted in Figs. 3 and 4, respectively; Besides, HI was separately determined for each brand and color; detailed data are given in Online Resource 1.

In most investigated brands, maximum levels of HQ were calculated for Pb and lowest levels were belonging to Fe. Moreover, Pb had the highest HQ value in different investigated colors according Fig. 4. Estimated HI showed that the most polluted brand and color are hair dye brand of code 2 (5.73E-4) and dark brown (1.93E-3), respectively.

### Sensitivity analysis

**Fig. 5:**
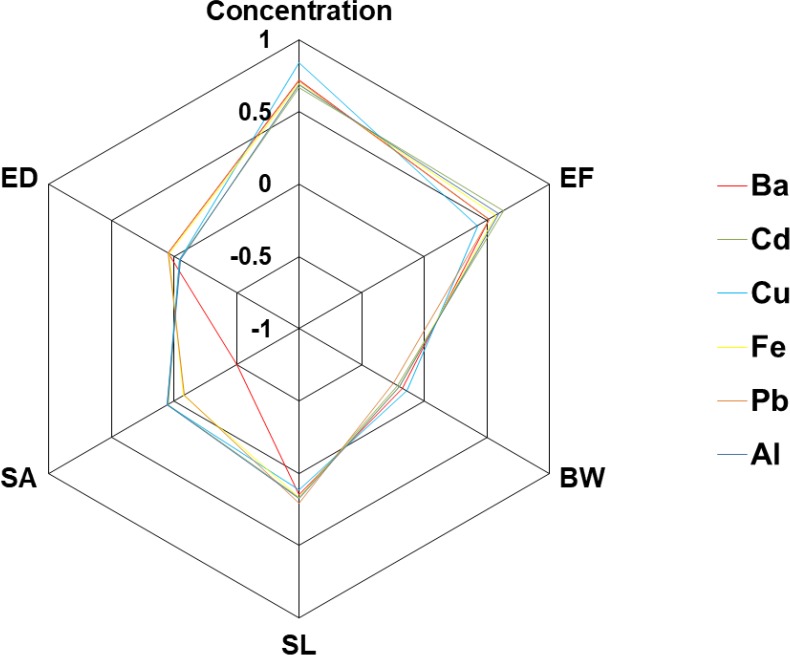
Sensitivity analysis bases on Rank Correlation

Sensitivity analysis was carried out to evaluate the rate of influence of different parameters (ED, SA, SL, BW, EF and concentration of element) on the D_dermal_ levels; for this purpose, rank correlation was calculated, the results are shown in Fig. 5.

## Discussion

Iran is the second biggest consumption market for cosmetic products in the Middle East (22). Chemical hair dyes are among the most common used cosmetic products in Iran and there are no studies about their contamination to heavy metals. In the current study, 32 samples of the most commonly used hair dyes in the Tehran markets were investigated for determining their contamination to some heavy metals; and subsequently, the non-carcinogenic health risk related to consumption of these products were evaluated.

### Concentration of Al

This metal causes Alzheimer’s disease and damages the nervous system in human body (23, 24). The concentration of this element ranged between 0.1 and 3.7 mg kg^−1^ in the investigated brands and colors; besides, the average of it was found 0.54±0.64 mg kg^−1^. Compared with other studies, the rate of this metal in all the collected samples was too low; for example, in Saudi Arabia on mascara and eye shades, the concentration of Al was reported as 50 mg kg^−1^ (25). In another study, this element was investigated in Henna and they found the concentration of Al as 142.1±1.52 mg kg^−1^ (14).

### Concentration of Ba

Ba is an alkaline metal replaced with Ca and deposited in bones. Furthermore, this metal causes vasoconstriction, hypertension, and toxicity in muscular systems (26, 27). We found that the average of this element in investigated samples was 0.86±1.43 mg kg^−1^ and this concentration was lower rather than the results of other studies. For example, in a research on different cosmetics, the rate of Ba in Henna was found 17.46 mg kg^−1^ and the range of this metal was between 10.37 and 1895 mg kg^−1^ in lipstick samples (14). The concentration of this metal was reported between 3.6 and 96.8 mg kg^−1^ in mascara and eye shades (28).

### Concentration of Cd

Exposure to Cd could affect the cardiovascular system and vessels (29). Moreover, this metal could threaten bones and it is toxic for cells and kidneys (7, 30, 31). The range of Cd concentration was found 0.0001–0.0012 mg kg^−1^ and also, the average of this element in the measured samples was 0.00045±0.0003 mg kg^−1^. In a study on different beauty products (lipstick, powder, kohl, cream, and shampoo), the average of this metal was reported as 0.000238±0.000001 mg kg^−1^ which it was lower than our findings (7). In another study, the concentration level of this metal in three products of cream, lipstick, and glosses lip was in range of 4 to 207 ppb (32). The concentration of Cd was found between 0.000625–0.0018 mg kg^−1^ in natural dye (Henna) (33).

### Concentration of Cu

Exposure to Cu could threaten the liver and create Wilson’s disease and insomnia (7). In addition, this metal with Cd could cause digestive disorders, diarrhea, tremor and ataxia, depression, and paralysis (34).

Average concentration of this metal in the analyzed samples was 61.32±100.59 ppb. This value was less than other findings (7, 14); they studied the concentration of metals in cosmetics; they determined the concentration of Cu 26.62 and 95.92 ppb, respectively (14). The range of this element was 141 to 37300 ppb in eyeshades and mascara (28). These levels were higher than the results of the current study.

### Concentration of Fe

We determined the average concentration of Fe in the investigated samples as 1.19 mg kg^−1^, and it was ranged from 0.17 to 11.58 mg kg^−1^ in different colors and brands. In compare to our findings, the average of Fe determined in hair pomade as 209.8 mg kg^−1^ (1). In another study about lipstick and eye shadow, it was reported as 20.3E+3 mg kg^−1^ and 10.2E+3 mg kg^−1^, respectively (10). Vomiting, dizziness, nausea, anorexia, headache, and weight loss are among some complications of Fe in the human body (7). Moreover, the high levels of Fe increase the potential risk of cancer through rapid catalysis of oxygen radicals in cells (35).

### Concentration of Pb

Pb is an unnecessary element for humans. Some poisoning symptoms of this element include loss of appetite, anemia, vomiting, hysteria, and autoimmune disease (36). Furthermore, this metal damages the central nervous system and can decrease the IQ of children (31). Premature birth and low birth weight are the other toxic effects of this element (32, 37). In present study, the measured concentration of this metal was equal to 0.00374 to 0.3855 mg kg^−1^, and average was determined as 0.185± 0.9049 mg kg^−1^. In a study on hair pomade, found the concentration of this element as 8.269 mg kg^−1^(1). Besides, this average was reported about 17.46 mg kg^−1^ in Henna samples (14). Our findings revealed that the level of Pb in investigated chemical hair dyes is lower than the Pb concentrations of cosmetic products in the other studies.

### Exposure assessment and risk characterization

Exposure assessment is one of the most important components of risk evaluation, used for investigating the probability and extent of exposure of people to a chemical agent (38). In current study, we surveyed the non-carcinogenic effect of investigated heavy metals through HQ calculation; according to Eq.3, If the value of HQ be obtained more than 1, it means that risk level is significant; if this index to be calculated lower than 1, the non-carcinogenic risk effect is not probable (15, 16, 19). All calculated HQ for different colors, brands were less than 1, and there was no significant health risk from investigated elements. Moreover, according to Eq.4, HI was calculated based on the obtained HQ_s_; similar to HQ, when the value of HI is more than 1 risk level will be significant. As can be seen in Online Resource 1, the calculated HI indices for the surveyed colors and brands were less than 1 which means, considering overall health risk of all surveyed elements, the consumption of different colors and brands of chemical hair dyes are safe, and the calculated HQ and HI had significant interval to the baseline of 1, this means the levels of human exposure to these elements cannot cause any deleterious effect.

### Sensitivity analysis

According to Fig. 5, the D_dermal_ values for the investigated elements were more affected by element concentration levels and after it, the parameter of exposure frequency is more correlated to the D_dermal_ values. Hence, the reducing of contamination of chemical hair dyes to the heavy metals is the most effective way to control the heavy metal exposure from chemical hair dyes consumption pathway.

## Conclusion

Two indices of HI and HQ showed that heavy metal contents in the investigated samples had not probable non-carcinogenic risks for the consumers of these products.

## Ethical considerations

Ethical issues (Including plagiarism, informed consent, misconduct, data fabrication and/or falsification, double publication and/or submission, redundancy, etc.) have been completely observed by the authors.
